# The Secretome Derived From 3D-Cultured Umbilical Cord Tissue MSCs Counteracts Manifestations Typifying Rheumatoid Arthritis

**DOI:** 10.3389/fimmu.2019.00018

**Published:** 2019-02-05

**Authors:** Joana P. Miranda, Sérgio P. Camões, Maria M. Gaspar, Joana S. Rodrigues, Manuela Carvalheiro, Rita N. Bárcia, Pedro Cruz, Helder Cruz, Sandra Simões, Jorge M. Santos

**Affiliations:** ^1^Faculty of Pharmacy, Research Institute for Medicines (iMed.ULisboa), University of Lisbon, Lisbon, Portugal; ^2^ECBio S.A., Amadora, Portugal; ^3^Centro de Estudos de Ciência Animal, Instituto de Ciências, Tecnologias e Agroambiente, Universidade do Porto, Porto, Portugal; ^4^Instituto de Tecnologia Química e Biológica António Xavier, Universidade Nova de Lisboa, Oeiras, Portugal

**Keywords:** mesenchymal stem/stromal cells, umbilical cord tissue, 3D culture, secretome, arthritic signs, rheumatoid arthritis

## Abstract

Rheumatoid arthritis (RA) is an autoimmune disorder whose treatment is mostly restricted to pain and symptom management and to the delay of joint destruction. Mesenchymal stem/stromal cells from the umbilical cord tissue (UC-MSCs) have previously been proven to be immunomodulatory and more efficient than bone marrow-derived MSCs in causing remission of local and systemic arthritic manifestations *in vivo*. Given the paracrine nature of UC-MSC activity, their application as active substances can be replaced by their secretome, thus avoiding allogeneic rejection and safety issues related to unwanted grafting. In this work, we aimed at demonstrating the viability of applying the 3D-primed UC-MSC secretome for the amelioration of arthritic signs. A proteomic analysis was performed to both, media conditioned by UC-MSC monolayer (CM2D) and 3D cultures (CM3D). The analysis of relevant trophic factors confirmed secretome profiles with very significant differences in terms of therapeutic potential. Whereas, CM3D was characterised by a prevailing expression of anti-inflammatory cytokines such as IL-10 and LIF, along with trophic factors involved in different mechanisms leading to tissue regeneration, such as PDGF-BB, FGF-2, I-309, SCF, and GM-CSF; CM2D presented relatively higher levels of IL-6, MCP-1, and IL-21, with recognised pro-inflammatory roles in joint disease and pleiotropic effects in the progression of rheumatoid arthritis (RA). Accordingly, different motogenic effects over mouse chondrocytes and distinct capacities of inducing glycosaminoglycan synthesis *in vitro* were observed between CM3D and CM2D. Finally, the evaluation of arthritic manifestations *in vivo*, using an adjuvant-induced model for arthritis (AIA), suggested a significantly higher therapeutic potential of CM3D over CM2D and even UC-MSCs. Histological analysis confirmed a faster remission of local and systemic arthritic manifestations of CM3D-treated animals. Overall, the results show that the use of UC-MSC CM3D is a viable and better strategy than direct UC-MSC administration for counteracting AIA-related signs. This strategy represents a novel MSC-based but nonetheless cell-free treatment for arthritic conditions such as those characterising RA.

## Introduction

The destruction and functional disability of joint tissues caused by arthritis impart a massive burden to health services worldwide. There are two basic types of arthritis: osteoarthritis (OA), a degenerative condition that is the result of increased wear and tear on joints, and autoimmune-based arthritis, such as rheumatoid arthritis (RA), which produces systemic inflammatory joint symptoms, with a greater incidence in synovial tissues. OA may also produce inflammatory symptoms, but primarily destroys joint cartilage over time. Neither form of arthritis has yet a treatment, which is able to reverse joint tissue wear out. The available alternatives are restricted to pain and symptom management and/or to prevent or delay further joint destruction. Efforts to discover new target therapies have achieved some success. However, these new approaches are very expensive and none of the currently widely used biological agents reaches long-term drug-free remission (1, 2). RA is a systemic disease and the mechanisms behind its symptoms are complex and have not yet been fully uncovered. The innate immune system, through activation of Toll-like receptors, contributes to a joint pathophysiology characterised by the recruitment of aberrant inflammatory cells, such as T-cell, B-cell, and macrophages, that together with periarticular factors, such as adipocytokines, cause chronic joint inflammation (3).

Given the lack of expression of MHC Class II, and residual expression of MHC Class I, mesenchymal stromal/stem cells (MSCs) are thought to have immune-privileged properties and as such may be delivered in the absence of HLA matching and/or immunosuppression (4–6). Additionally, MSCs have immunomodulatory properties and as such have been successfully applied for the treatment of inflammatory and immune-mediated adverse reactions, such as graft vs. host disease (GVHD), organ rejection after transplantation, allergy, and autoimmune diseases (7–11).

Autoimmune-driven joint destruction, caused by persistent inflammation, renders RA a possible clinical target for cartilage and bone repair using MSCs (12). Indeed, previous results, using a mouse adjuvant-induced model for arthritis (AIA), showed that autologous bone marrow-derived MSCs (BM-MSCs), injected in the joints were able to reduce joint swelling and cartilage destruction, by decreasing the levels of TNF-α. These cells were shown to integrate into the synovium (13). More recently, conditioned medium from the same BM-MSCs was used for treatment (with increased levels of IL-10) significantly reducing histopathological signs of AIA, cartilage damage and suppressing immune responses by reducing aggrecan cleavage, enhancing Treg function and adjusting the Treg:Th17 ratio (14).

Alternatively to BM-MSCs, MSCs from the umbilical cord stromal tissue, or Wharton's jelly (UC-MSCs), can be safely used for allogeneic approaches given their lack of immunogenicity and their marked capacity for localised immunosuppression (15). This immunomodulatory effect is not contact-dependent and is thought to be mostly due to secreted paracrine factors. In addition, UC-MSCs have advantages over other MSCs given their relatively easy and non-invasive procurement, higher expansion potential and overall higher potency to differentiate into more diverse specialised cells originating from the three germ layers (16, 17).

In our previous studies we have shown that the immunomodulatory properties of a particular population of human UC-MSCs, when compared to human BM-MSCs, were less immunogenic, suitable for xenotransplantation without inducing immunologic infiltrates, and had higher immunosuppression activity than BM-MSCs. Furthermore, unlike BM-MSCs, UC-MSCs did not need prior activation or priming to exert their immunomodulatory effects *in vivo*. Several gene and protein expression profile differences were found between UC-MSCs and BM-MSCs that could explain such observations, namely the increased expression of immunomodulatory surface proteins such as CD200, CD273, CD274, and cytokines such as IL-1β, IL-8, LIF, and TGF-β2 by UC-MSCs (6). In another comparative study, this time in the context of cutaneous wound healing, UC-MSCs have shown to secrete considerably higher amounts of G-CSF, EGF, FGF-2, and KGF than BM-MSCs, with concomitant improved motogenic effects over keratinocytes and fibroblasts, as well as enhanced pro-angiogenic activity (18). Accordingly, UC-MSCs were shown to potentially induce the regenerative capacity of tissues *in vivo* by attracting other endogenous MSCs via a unidirectional, UC-MSC-specific, G-CSF-mediated mechanism (18). More relevant to this study, such population of UC-MSCs when transplanted *in vivo*, in a rat AIA model, showed to be non-immunogenic, to have immunosuppressive properties through the inhibition of T-cell proliferation and induction of Tregs, and to promote an impressive remission of local and systemic arthritis manifestations (19, 20). Interestingly, by taking advantage of the 3D greater cell-to-cell communication and cell-to-extracellular matrix (ECM) interactions, the same cells cultured as self-aggregated spheroids could be primed toward a better therapeutic phenotype, as demonstrated in a wound healing context (21).

In this work, we aimed at demonstrating the feasibility of applying a 3D-culture-based UC-MSC priming strategy to improve the efficacy of the resulting secretome for the treatment of inflammatory arthritis. Differences in proteomic profiles and *in vitro* and *in vivo* therapeutic potentials were confirmed between secretomes produced in either 3D spinner flask bioreactors or UC-MSCs cultured under conventional two-dimensional (2D) monolayer conditions. The results clearly showed an improved efficacy of a UC-MSC 3D-derived secretome for the amelioration of experimental AIA manifestations, even when compared with the direct administration of UC-MSCs. The potential mechanisms behind our observations are discussed, as we disclose the potential use of a UC-MSC 3D-primed secretome, or some of its components, as active substances for *Advanced Therapy Medicinal Products* (ATMP) for the treatment of RA.

## Materials and Methods

### Reagents

Antibodies and their respective isotypes were acquired from BioLegend (San Diego, CA, USA) unless stated otherwise. Cell culture media and supplements were purchased from Sigma-Aldrich (Madrid, Spain), unless otherwise stated. FBS, Formalin, Trypsin/EDTA (ethylenediamine tetra-acetic acid), Eosin Y and Harris's haematoxylin: Gibco (Life Technologies, Madrid, Spain). BCA protein assay kit: Novagen (San Diego, CA, USA). Blyscan™ Sulfated Glycosaminoglycan Assay kit: Biocolor (Carrickfergus, UK). Entellan®: Merck (Darmstadt, Germany). Flow-Cytomix™: eBioscience. TGF-β1: Tebu-bio (Le-Parray-en-Yvelines, France). Tissue Tek® O.C.T.™: Sakura (Zoeterwoede, The Netherlands). Xylene: EMD Chemicals, Inc. (Gibbstown, NJ, USA).

### UC-MSC Isolation and Culture

#### UC-MSC Isolation

This study was approved by the Ethics Committee of the Hospital Dr. José de Almeida (Cascais, Portugal), in the scope of a research protocol between ECBio (Research & Development in Biotechnology, S.A.) and HPP Saúde (Parcerias Cascais, S.A.). Umbilical cord donations, with written informed consents, as well as umbilical cord procurement, were made according to Directive 2004/23/EC of the European Parliament and of the Council of 31 March 2004 on setting standards of quality and safety for the donation, procurements, testing, processing, preservation, storage, and distribution of human tissues and cells. UC-MSCs were isolated from umbilical cords of healthy new-born babies, upon informed consent of healthy parturients, as previously described (22). Cells were cryopreserved in minimum essential medium Eagle alpha modification (α-MEM) containing 10% dimethyl sulfoxide (DMSO) stock solution and 20% foetal bovine serum (FBS), using a controlled rate of temperature decrease. When needed, UC-MSCs cryopreserved between passage 3 (P3) and P5 were thawed and further expanded during a maximum of 30 cumulative population doublings (cPDs), corresponding to P12 in culture. UC-MSCs are known to undergo at least 55 cPDs (P22) before reaching senescence, keeping MSC phenotype (21).

#### Three-Dimensional (3D) Culture

For 3D cultures, spinner vessels (125 mL) with ball impeller containing α-MEM supplemented with 15% FBS were inoculated with single cell suspensions at a concentration of 1 × 10^6^ cells/mL. To promote cell aggregation spinner vessels were stirred at 80 rpm and kept at 37°C in a humidified atmosphere of 5% CO_2_ for 24 h. After this period, half of the cell culture supernatant was replaced by fresh medium supplemented with 10% FBS (v/v). Culture medium was replaced every 3–4 days and the stirring rate was adjusted to 110 rpm to maintain spheroid size below 350 μm.

#### Two-Dimensional (2D) Monolayer Culture

For two-dimensional (2D), static monolayer, cultures, cells were seeded at a density of 1 × 10^4^ cells/cm^2^ in α-MEM supplemented with 10% FBS and incubated at 37°C in a humidified atmosphere with 5% CO_2_. Cell passage was performed by Trypsin/EDTA 0.05% incubation for 5 min every 72 h.

### UC-MSC Characterisation

#### Flow Cytometry

Cell surface marker expression was analysed by flow cytometry in both 2D and 3D cultures. Cell detachment from culture t-flasks and dissociation from spheroids was performed by using 0.25% Trypsin/EDTA. The resulting single cell suspension was washed with 2% bovine serum albumin (BSA) in phosphate-buffered saline (PBS). Detection of cell surface markers was performed with the following antibodies and their respective isotypes after incubation for 1 h at 4°C: phycoerythrin (PE) anti-human CD105 (eBioScience, San Diego, CA, USA); APC anti-human CD73; PE antihuman CD90; APC anti-human CD44; PerCP/Cy5.5 anti-human CD45; fluorescein isothiocyanate (FITC) anti-human CD34; FITC anti-human CD31; PerCP/Cy5.5 anti-human CD14; Pacific Blue anti-human CD19 and pacific-blue anti-human HLA-DR. All samples were acquired on a Gallios (Beckman Coulter, Pasadena, CA, USA) and the results analysed with Kaluza software (Beckman Coulter). A minimum of 1 × 10^4^ events were acquired per surface marker. One replicate was analysed per independent experiment (*n* = 4).

#### Tri-lineage Differentiation

Spheroids were dissociated into a single cell suspension with 0.25% Trypsin/EDTA and transferred to appropriate culture t-flasks for cell proliferation and expansion. To induce adipogenic differentiation, UC-MSCs were incubated in α-MEM supplemented with 20% FBS, 10 μg/mL insulin, 200 μM indomethacin, 0.5 mM isobutylmetylxantine, and 1 μM dexamethasone for 3 days and 1 day in medium supplemented with 20% FBS and 10 μg/mL insulin. To induce osteogenic differentiation, cells were incubated in α-MEM supplemented with 10% FBS, 10 mM β-glycerol phosphate, 100 nM dexamethasone, and 50 μg/mL ascorbate-2-phosphate. Finally, to induce chondrogenic differentiation, cells were maintained in suspension as pellets, incubated with Dulbecco's modified Eagle's medium (DMEM) with 4 mM glutamine and 1 g/L D-(+)-glucose, supplemented with 1% FBS, 6.25 μg/mL insulin, 10 ng/mL transforming growth factor (TGF)-β1, and 50 μM ascorbate-2-phosphate. For cytochemical staining, cells were fixed with paraformaldehyde 4% for 20 min. In adipogenic and osteogenic differentiation protocols, cells were stained with Oil Red O for 10 min and alkaline phosphatase for 30 min, respectively. For chondrogenic differentiation, the chondrospheres were fixed in formalin, embedded in paraffin and cut into sections of 5 μm and stained with alcian blue for 30 min. The presence of stained cells was confirmed by inverted microscopy with phase contrast (Leica, DMIL HC, Wetzlar, Germany).

#### Protein Quantification

For both 3D and 2D cultures, biomass was evaluated by total protein quantification using a BCA protein assay kit, after cell pellet lysis with 0.1 M NaOH at 37°C for 24 h.

#### UC-MSC Spheroid Visualisation and Measurement

Spheroids were observed by bright field microscopy (Olympus CK30, Olympus, Tokyo, Japan) and their average diameter determined by a geometric mean of three diameters per spheroid as previously described, using the following equation: average diameter = (d1 × d2 × d3)^1/3^ (17, 23). Diameters were measured using Motic Images Version 2.0 software (Xiamen, China).

#### Haematoxylin and Eosin Staining

Spheroids were suspended in Tissue Tek® O.C.T.™ for preparing 10 μm cryosections. Slides were first stained with Harris's haematoxylin for 10 min, followed by incubation with HCl 1% (v/v) in 70% EtOH, and by Eosin Y staining for 2 min. Slides were then submitted to increasing concentrations of ethanol and finally incubated in xylene. Samples were mounted with Entellan®. Images were acquired on an Olympus CK30 inverted microscope and processed using Motic Images Version 2.0 software.

### Conditioned Media (CM) Preparation

Conditioned media (CM) were produced from cells having undergone the same number of cPDs. UC-MSC CM from 3D spinner flask cultures (CM3D) was obtained by cell inoculation as described above, subjected to successive medium adaptations: FBS concentration was reduced to 5% at culture day 2. After 3 days, medium was changed with α-MEM without FBS and volume adjusted to obtain a conditioning volume per cell equivalent to that in the 2D system. After 48 h of conditioning, CM3D was collected under sterile conditions. To produce UC-MSC CM in 2D monolayer cultures (CM2D), 1.75 × 10^6^ cells were seeded in 175 cm^2^ culture t-flasks and kept in medium supplemented with 5% FBS until they reached 90% confluence. At this point, cells were washed with fresh α-MEM and medium was replaced by α-MEM without FBS, to a final volume of 25 mL. After conditioning for 48 h, CM2D was harvested under sterile conditions. The control sample consisted of α-MEM which was never in contact with cells. CM3D, CM2D, and control were 10 × concentrated using 3-kDa cut-off spin concentrators. Total protein content of CM2D, CM3D and control was quantified using a BCA protein assay kit. Samples were stored at −80°C until further use.

#### CM2D and CM3D Trophic Factor Quantification

Trophic factor concentrations within CM3D and CM2D samples were measured using the Human 64-Plex Cytokine/Chemokine Panel (Eve Technologies, Calgary, AB, Canada) or using Flow-Cytomix™ according to manufacturer's recommendations. All cytokines/chemokines, except for IL-6 and MCP-1, were quantified by resorting to the multiplexing technology. IL-6 and MCP-1 detections were acquired on a Gallios (Beckman Coulter) and the results were obtained using FlowCytomix™ Pro 3.0 Software. Data is expressed in terms of productivity: ng/mL/million cells/hour, normalised against the background (α-MEM that was never in contact with cells) threshold concentrations.

### CM Testing *in vitro*

#### Mouse Chondrocyte (ATDC5) Cell Culture

Mouse chondrocytes (ATDC5) were seeded at 1 × 10^4^ cells/cm^2^ and cultured in DMEM-F12 supplemented with 5% FBS, at 37°C, in 5% CO_2_ humidified atmosphere until reaching 70–80% confluence. Cell passage was performed by Trypsin/EDTA 0.25% incubation for 5 min every 72 h.

#### Scratch Assay

ATDC5 cells were seeded into 24-well plates at a density of 1.5 × 10^4^ cells/cm^2^ with DMEM-F12 supplemented with 5% FBS. Once at 90% confluence, scratches of ~0.5 mm in width were performed on the monolayer with a sterile 200 μL pipette tip. Immediately after, the cell surfaces were washed with PBS and maintained in a final volume of 400 μL of DMEM-F12 supplemented either with CM2D, CM3D, all 10 × concentrated. DMEM-F12, DMEM-F12:α-MEM (1:1) and DMEM-F12 with 5% FBS were also tested as negative, solvent and positive controls, respectively. The area of the scratch, from the same field, was measured at 0, 3, 6, 8, 10, 20, and 24 h post-scratch to evaluate cell migration. Digital photographs were taken at an amplification of 40 × on Olympus CK30 microscope. Cellular migration was analysed in the Motic Images Version 2.0 software by calculating scratch closure, given as the total area occupied by the cells after incubation with CM in relation to the initial scratch area at 0 h. Three independent experiments in triplicates were considered.

#### Glycosaminoglycan Quantification

Glycosaminoglycans (GAG) were quantified in ATDC5 cell culture supernatants. At a confluence of 60%, cells were incubated with DMEM-F12 supplemented either with CM2D or CM3D 10 × concentrated. DMEM-F12, DMEM-F12:α-MEM (1:1) and DMEM-F12 with 5% FBS were also tested as negative, solvent and positive controls, respectively. After 24 h of incubation, GAGs were determined using the Blyscan™ Sulfated Glycosaminoglycan Assay kit, according to the manufacturer's instructions. A total of three independent experiments were performed.

### CM Testing *in vivo*

#### Adjuvant-Induced Arthritis (AIA) Model

All animal experiments were carried out with the permission of the local animal ethical committee in accordance with the EU Directive (2010/63/EU), Portuguese law (DL 113/2013) and all relevant legislations. The experimental protocol was approved by *Direcção Geral de Alimentação e Veterinária* (DGAV). Animals were acclimatised before the experiments and housed in plastic cages under standard laboratory conditions, fed commercial chow, and acidified drinking water *ad libitum*.

Induction of the inflammatory reaction was achieved by injecting Wistar rats (365–480 g; Charles River Laboratories, France) with a single intradermal (i.d.) administration of 0.1 mL of a suspension of killed and dried *Mycobacterium butyricum* in incomplete Freund's Adjuvant—IFA (at 10 mg/mL) (Difco Laboratories), into the sub-plantar area of the right hind paw (24). Animals were randomly divided into groups (*n* = 3-6). Treatment was initiated at day 7 after induction. Induced animals were treated with different formulations: (i) one group received PBS by intraperitoneal (i.p.) injection (Sham UC-MSC group); (ii) another group received PBS by intra-articular (i.a.) route of administration (Sham CM group); (iii) a third group received 2D-cultured UC-MSCs (1.7 × 10^6^ cells per injection in 4 consecutive days) by i.p. injection (UC-MSC group); (iv) a fourth group received, by i.a. injection, 10 × concentrated CM2D (CM2D group); (v) one group received, by i.a. injection, 10 × concentrated CM3D (CM3D group); and lastly, (vi) three animals received neither inflammation induction nor any treatment and were used as a naïve control for histology analysis (Control). No experimental group received 3D-cultured UC-MSCs since full cell disaggregation from 3D aggregates has proven very difficult with consequent risk of acute inflammation and thrombosis. Animals treated intraperitoneally and the respective control group (Sham UC-MSC group) received a volume of 100 μL of the tested formulations per injection (i.e., a total of 400 μL). Animals treated intrarticularly and the respective control group (Sham CM group) received a volume of 200 μL of the tested formulations per injection (i.e., a total of 600 μL). I.p. injections were given for 4 consecutive days. I.a. administrations, in a total of three, were performed every-other-day. The experiment lasted for 57 days. To follow the course of the disease, body weight and volume of right and left paws measured by a water displacement method, using a plethysmometer (Ugo Basile, Italy), were measured. Blinding investigators to treatment groups were defined. Arthritis was evaluated in ankle joints in a blinded manner using a semiquantitative arthritic score based on the sum of the following grades: 0 = normal; 1 = for each inflamed paw; 1 = tail lesion; 1 = joint rigidity or deformity; 1 = wounded paw; 1 = infected paw; 1 = necrotic paw. The sum of the parameters is calculated as an arthritic index (AI) with a maximum possible score of 9. Photographs recorded the evolution of clinical signs in all experimental groups. Animals were sacrificed at day 57, necropsies and gross pathology examination was conducted. The experiment was performed twice with consistent observations, using two UC-MSC isolates (different donors).

#### Collection of Paw Samples and Histopathological Analysis

After the sacrifice, animal paws were collected, fixed in 10% buffered formalin and decalcified with 10% formic acid to undergo histopathological analysis. In order to evaluate the surroundings of the site of application, fixed decalcified paws were processed for embedding in paraffin wax by using routine protocol. Sections (5 μm thick) were stained with haematoxylin and eosin (H&E). The slides were examined using light microscopy using an Olympus BX 40 microscope coupled with an Olympus DP 10 camera (Olympus, Shinjuku, Tokyo, Japan). Digital photographs were taken at an amplification of 100×, except for the control that was acquired at an amplification of 40×. The histological samples were evaluated for synovial inflammation and bone erosion. Synovial inflammation was scored as follows: 0- no inflammation; 1- slight synovitis with some cell infiltration; 2- moderate synovitis with moderate cell infiltration; 3- extensive synovitis with a moderate number of infiltrating cells; 4- extensive and severe synovitis, with the presence of numerous inflammatory cells. Bone erosion was scored as follows: 0- no erosion; 1- small areas of resorption; 2- numerous areas of resorption; 3- extensive osteolysis; 4- extensive and severe osteolysis.

### Statistical Analysis

Statistical analyses were performed in GraphPad Prism v6.0 software (La Jolla, CA, USA). To estimate the significance of the differences of trophic factor quantification and of the data obtained from GAG production *in vitro*, multiple *t*-tests and Student's paired *t*-test with one-tailed distribution were used, respectively. The two-way ANOVA with Tukey's *post-hoc* test was performed for the *in vitro* scratch assay data. Results are presented as means ± standard error of the mean (SEM), except where indicated and *p*-values are presented for statistically significant results (^*^p < 0.05, ^**^p < 0.01, and ^***^p < 0.001).

## Results

### Pre-conditioning UC-MSCs in Tri-dimensional (3D) Culture Conditions Results in a Secretome Richer in Therapeutically Relevant Trophic Factors

**Figure 1:**
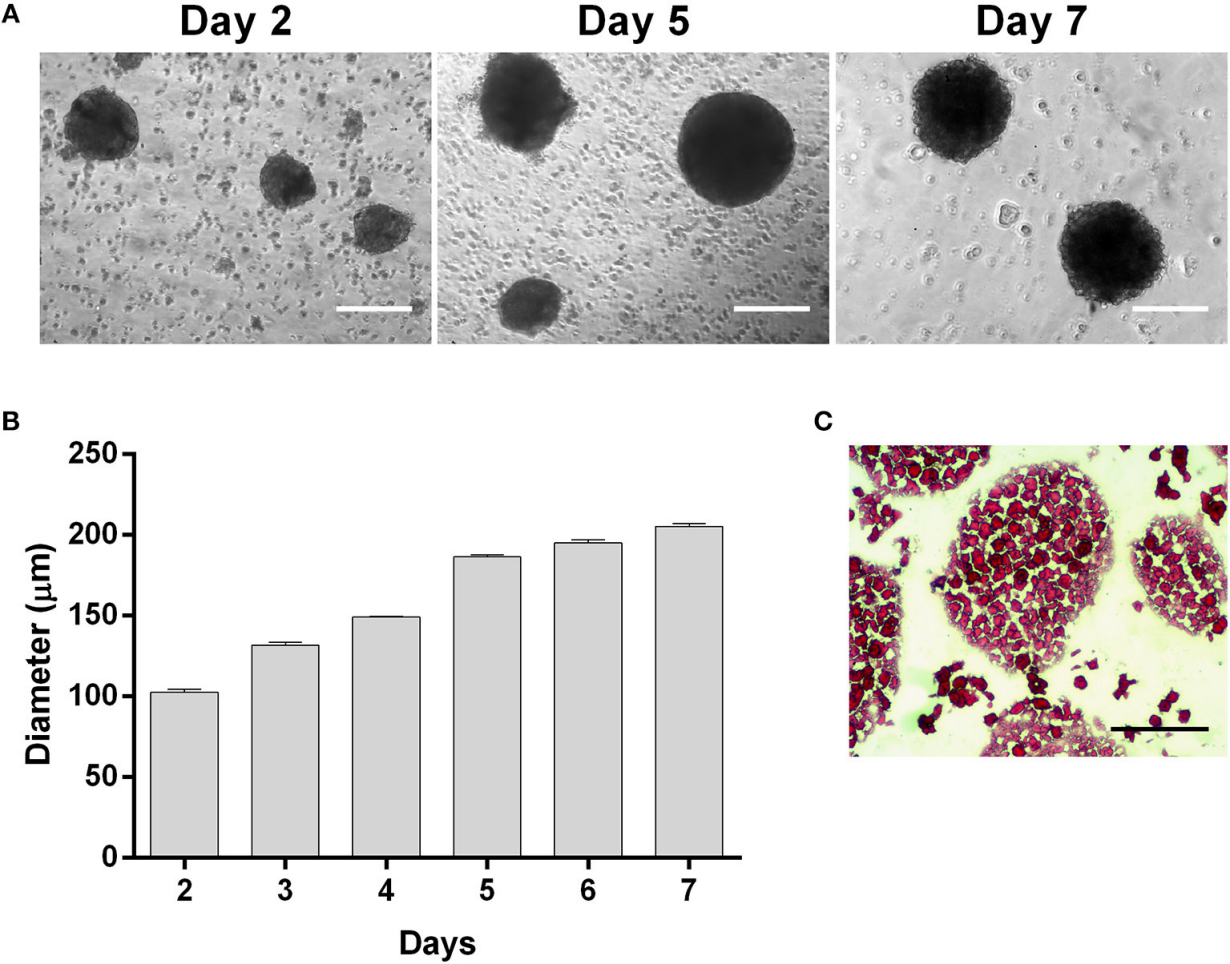
UC-MSCs form viable three-dimensional spheroids. **(A)** Representative phase contrast images of UC-MSC spheroids from days 2, 5 and 7 of culture. **(B)** UC-MSC spheroid size during culture time. **(C)** Representative image of UC-MSC spheroid sections from day 7 of culture stained with H&E. Scale bar = 100 μm. *n* = 7.

Three-dimensional (3D) spheroids from UC-MSCs were obtained using a spinner flask suspension culture. Measurements of spheroids were performed by phase-contrast images throughout the whole culture period yielding the size-distribution plot shown in Figure 1. Firstly, UC-MSCs form small low-density cell aggregates of ~100 μm diameter (Day 2, Figures 1A,B). After 4 days, spheroid diameters were, on average, 149.11 ± 0.57 μm and 195.48 ± 5.48 μm from day 5 to 7 of culture (Figures 1A,B). The results showed that the formation of more dense and viable 3D structures from day 4 onwards, with expected low diffusion rate of nutrients (Figures 1B,C). Nevertheless, a necrotic centre in spheroids was circumvented by maintaining the average spheroid size under 350 μm (Figure 1C).

**Figure 2:**
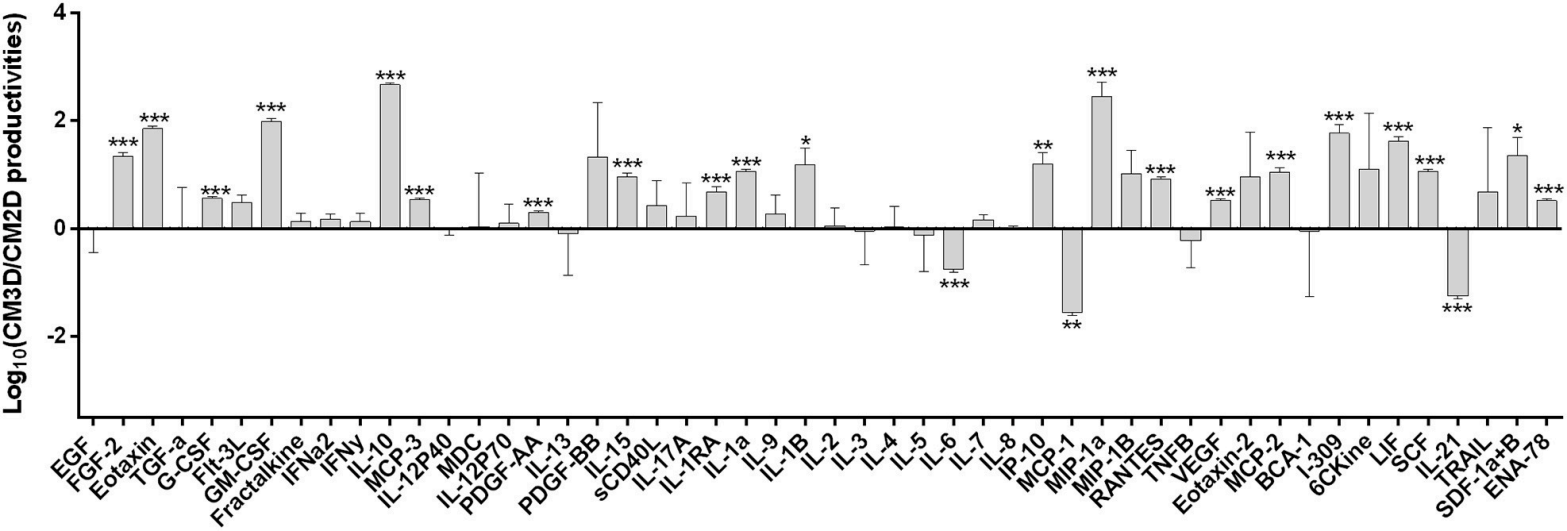
UC-MSCs display differences in specific trophic factor productivities that could entail different therapeutic activities between CM3D and CM2D. Quantification in CM3D and CM2D of a representative pool of trophic factors involved in relevant immune-modulation and other RA-relevant regenerative cascades of events. Data is expressed as the logarithm of the ratio between CM3D and CM2D productivities (ng/mL of the growth factors or cytokines/million cells/hour) corrected by subtracting the background (α-MEM). Plotted ratios are mean ± SD. *n* = 3. ^*^p < 0.05, ^**^p < 0.01, and ^***^p < 0.001.

In the present work, we firstly verified if the 3D culture conditions prompted the production of a secretome with higher potential to counter AIA signs than that obtained by UC-MSCs grown under 2D monolayer conditions. A comparative analysis of a representative pool of trophic factors involved in relevant immune-modulation and other relevant joint tissue regeneration events was performed between CM3D and CM2D. The relative productivity of such trophic factors was determined taking into consideration cell populations kept in similar conditions in terms of number of cells (with equivalent number of cPDs), same medium volume and conditioning time. The only variable in the experimental design was therefore the culture setup for modulating cell phenotype: 2D vs. 3D. Figure 2 shows the logarithm (Log_10_) of CM3D/CM2D ratio, representing the relative trophic factor productivity in 3D vs. 2D culture conditions normalised against the background medium that was never in contact with cells (α-MEM).

The results depicted in Figure 2 clearly show some interesting differences in specific trophic factor productivities that could result different therapeutic activities between CM3D and CM2D. CM3D was characterised by a prevailing expression of anti-inflammatory cytokines such as IL-10 and LIF, as well as trophic factors involved in different mechanisms leading to tissue regeneration, mainly PDGF-BB, FGF-2, I-309, SCF, and GM-CSF (25–29). In turn, CM2D was characterised by relatively higher levels of cytokines with recognised pleiotropic roles in the progression of inflammatory arthritis, such as IL-6, MCP-1, and IL-21 (30–32). To confirm if the differences observed in trophic factor profiles would indeed translate into different paracrine activities *in vitro*, we set forward to evaluate (i) the relative capacity of CM3D and CM2D to induce motility of joint chondrocytes and (ii) the relative capacity of CM3D and CM2D to induce glycosaminoglycan (GAG) synthesis, two important mechanisms connected to joint regeneration and arthritic aetiology.

### CM3D Has a Higher Motogenic Activity Over Mouse Chondrocytes *in vitro*

**Figure 3:**
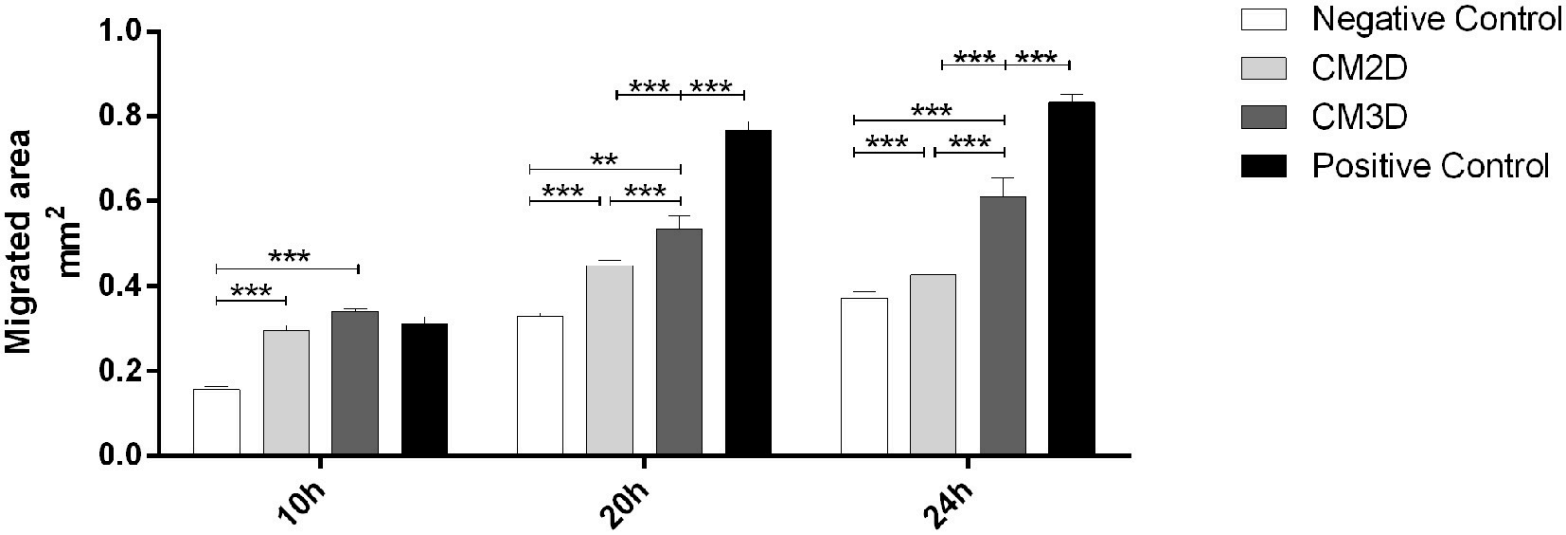
CM3D has a higher motogenic activity over mouse chondrocytes *in vitro*. Graphical representation of the percentage of reduction in the scratched area of mouse chondrocytes (ATDC5) evaluated through *in vitro* scratch assay after 10 h, 20 h and 24 h. ATDC5 cells were maintained in DMEM-F12 (negative control) or DMEM-F12 supplemented either with CM2D, CM3D or 5% FBS (positive control). *n* = 3. ^**^p < 0.01, and ^***^p < 0.001.

The relative capacity of CM3D and CM2D to promote chondrocyte migration was evaluated by scratch assays. Scratch areas were monitored for 24 h post-scratch. The results depicted in Figure 3 confirm significant differences between the paracrine activities of CM3D and CM2D. The CM3D supplement promoted a ~1.5-fold increase in chondrocyte migration capacity 24 h post-scratch when compared to CM2D, a fact that could be explained by e.g., relatively higher CM3D expression of PDGF-BB, IL-10, and FGF-2, all with recognised mitogenic, protective, and motogenic activities over chondrocytes (33–35).

### CM2D Has a Higher Capacity of Inducing Glycosaminoglycan (GAG) Synthesis *in vitro*

**Figure 4:**
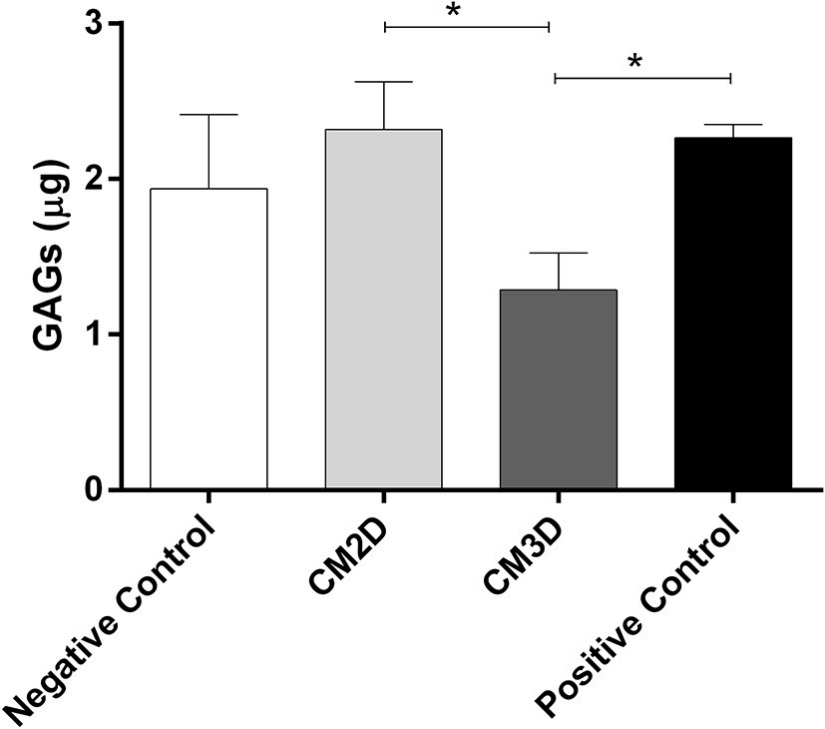
CM2D has a higher capacity of inducing glycosaminoglycan (GAG) synthesis *in vitro*. GAG production by mouse chondrocytes (ATDC5) was quantified in cell culture supernatant from cultures maintained in DMEM-F12 (negative control) or DMEM-F12 supplemented either with CM2D, CM3D, or 5% FBS (positive control). *n* = 3. ^*^p < 0.05.

Differences between the paracrine activities of CM3D and CM2D were further confirmed *in vitro* by evaluating their relative capacities to induce GAG synthesis. GAG concentration was quantified in the supernatant after 24 h of incubation. The results depicted in Figure 4 clearly show a ~2-fold increase in GAG induction by CM2D when compared to CM3D, a fact that could be explained e.g., by the relatively higher CM2D expression of IL-6 which has been shown to stimulate fibroblastic GAG synthesis and chondrocyte cartilage matrix production *in vitro* (36, 37).

### CM3D Has a Higher Capacity for Both, Avoiding and Ameliorating Adjuvant-Induced Arthritis (AIA) Manifestations *in vivo*

Given the *in vitro* evidence that CM3D and CM2D had in fact different paracrine activities regarding important mechanisms connected to joint regeneration and arthritic aetiology, we set out to evaluate the potential benefits of UC-MSCs primed by 3D culturing for reverting AIA signs *in vivo*.

The AIA model is useful for addressing the protective effects against manifestations typifying the late stage of entrenched chronic arthritis, sharing several pathological features with RA. The model is established by injecting *Mycobacterium butyricum* in incomplete Freund's Adjuvant into the sub-plantar area of Wistar rats' right hind paws. In our well-characterised setup, the disease shifts from a local arthritis stage to a systemic polyarthritis condition by day 13 after induction (20, 38). Indeed, we previously refined and characterised AIA by studying the time course of the disease, introducing new evaluation methods and identifying the main stages of the disease (24). One day after the disease induction, the induced paw volume more than doubled and haematological parameters completely changed, corresponding to the first disease phase. Two weeks after induction, another stage occurred when the disease shifted from the local arthritis form toward a systemic polyarthritis along with an additional increase of the paw volume (20, 38). Animal body weight also reached the minimum values and radiographic observable joint lesions increased accordingly (24). Starting the treatment at day 13 or later hampers the possibility to stop and reverse joint erosion (data not shown). Early recovery on body weight was obtained for animals treated at day 7 when compared with animals treated later (data not shown). As such, our treatment protocol started at day 7 post-induction and lasted for 57 days. No adverse effects were observed during or after the treatment period.

I.a. administration of CM3D and CM2D was performed and compared to the better performing route for UC-MSC administration, which had been found previously to be i.p. in the same AIA model (20). No experimental group received 3D-cultured UC-MSCs since full cell desegregation from 3D aggregates has proven very difficult with consequent risk of acute inflammation and thrombosis. The body weight, inflammatory swelling, clinical scoring through arthritic index (AI), and histopathological endpoints were measured.

**Figure 5:**
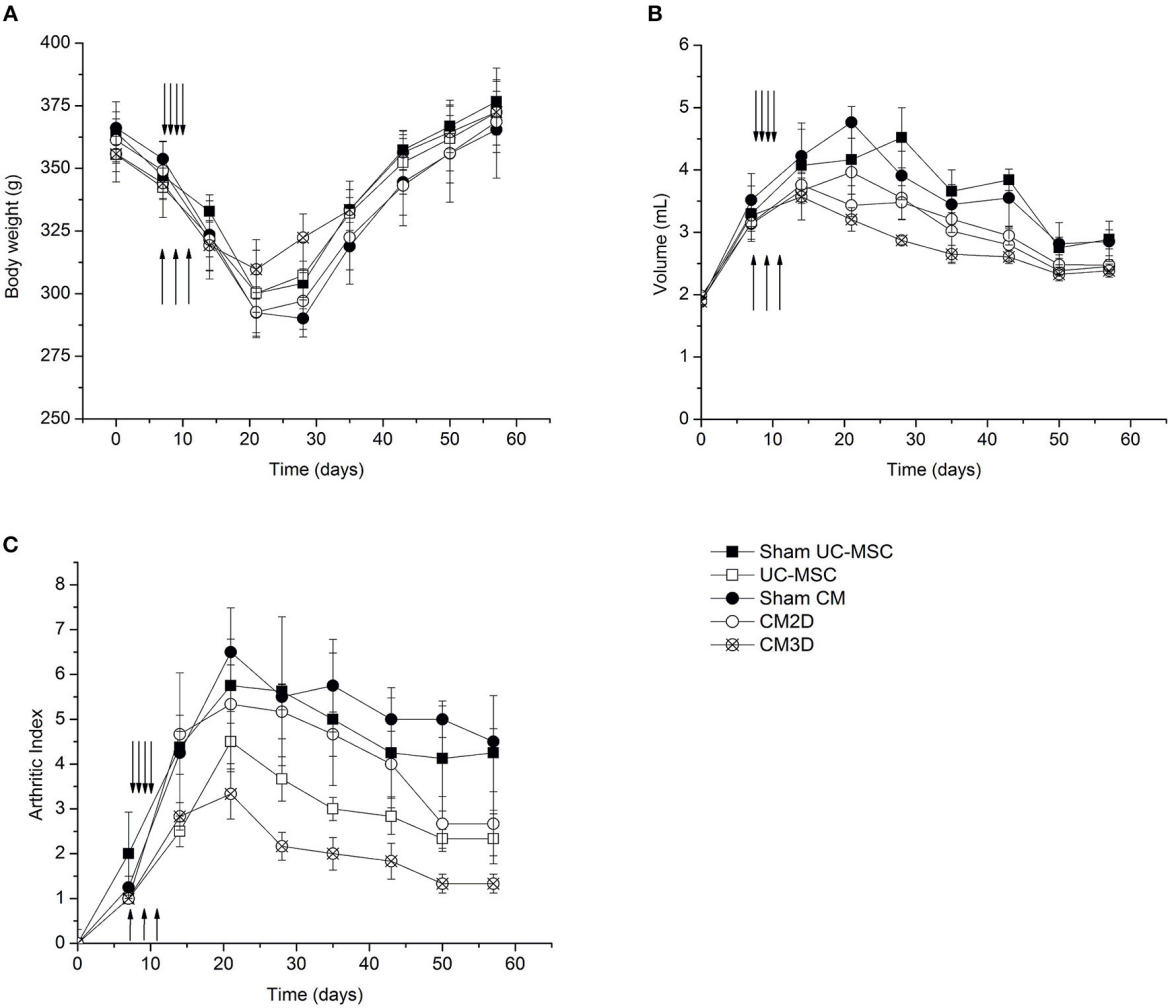
CM3D ameliorates adjuvant-induced arthritis (AIA) manifestations *in vivo*. The course of the disease was followed by measuring the **(A)** body weight, **(B)** the volume of the right paw (induced paw) and by **(C)** quantifying the disease score (arthritic index) of UC-MSC-, CM3D-, and CM2D-treated animals, and corresponding controls (Sham). Upward arrows represent the days of i.a. injections (CM administration). Downward arrows represent the days of i.p. injections (UC-MSC administration). *n* = 4 for sham groups; *n* = 6 for all other groups.

The time course of body weight is illustrated in Figure 5A. Given the transient nature of the AIA model, animals in all groups ultimately recovered from AIA manifestations and regained their natural body weights. However, weight loss was less prominent in animals treated with CM3D, especially between days 20 and 30 where the arthritic signs reached their highest intensity.

The hind paw volume was monitored by water displacement as a function of time after disease induction (Figure 5B). Swelling of adjuvant-injected right paws with erythema became evident within 1 day after induction. As seen in Figure 5B, animals from all treatment groups (UC-MSC, CM3D, and CM2D) showed ameliorated swelling. Nevertheless, the effect elicited by CM3D, as reflected by a considerable swelling reduction rate from day 15 onwards, conveys an unprecedented capacity for concomitantly prevent against the implantation and revert AIA inflammatory signs *in vivo*.

The development course of AIA signs was also evaluated by monitoring the evolution of the arthritic index (AI) as a function of time after disease induction (Figure 5C). All UC-MSC-based treatments resulted in amelioration of AIA manifestations when compared to the Sham controls as seen at day 20. Notably, treatment with CM3D resulted in more than a 2-fold reduction in AIA severity at day 20, culminating in a 5-fold difference at day 57, relatively to untreated animals. When compared to CM2D, CM3D was able to prevent the development of the AI more efficiently by a factor of ~1.8-fold at day 20, and still culminating with a 1.5-fold amelioration at day 57. The amelioration effect of CM3D was not so pronounced when compared to the administration of UC-MSCs at day 20 (1.5-fold), although in the end the amelioration effect brought by CM3D was still significant throughout the full experimental time frame (Figure 5C).

**Figure 6:**
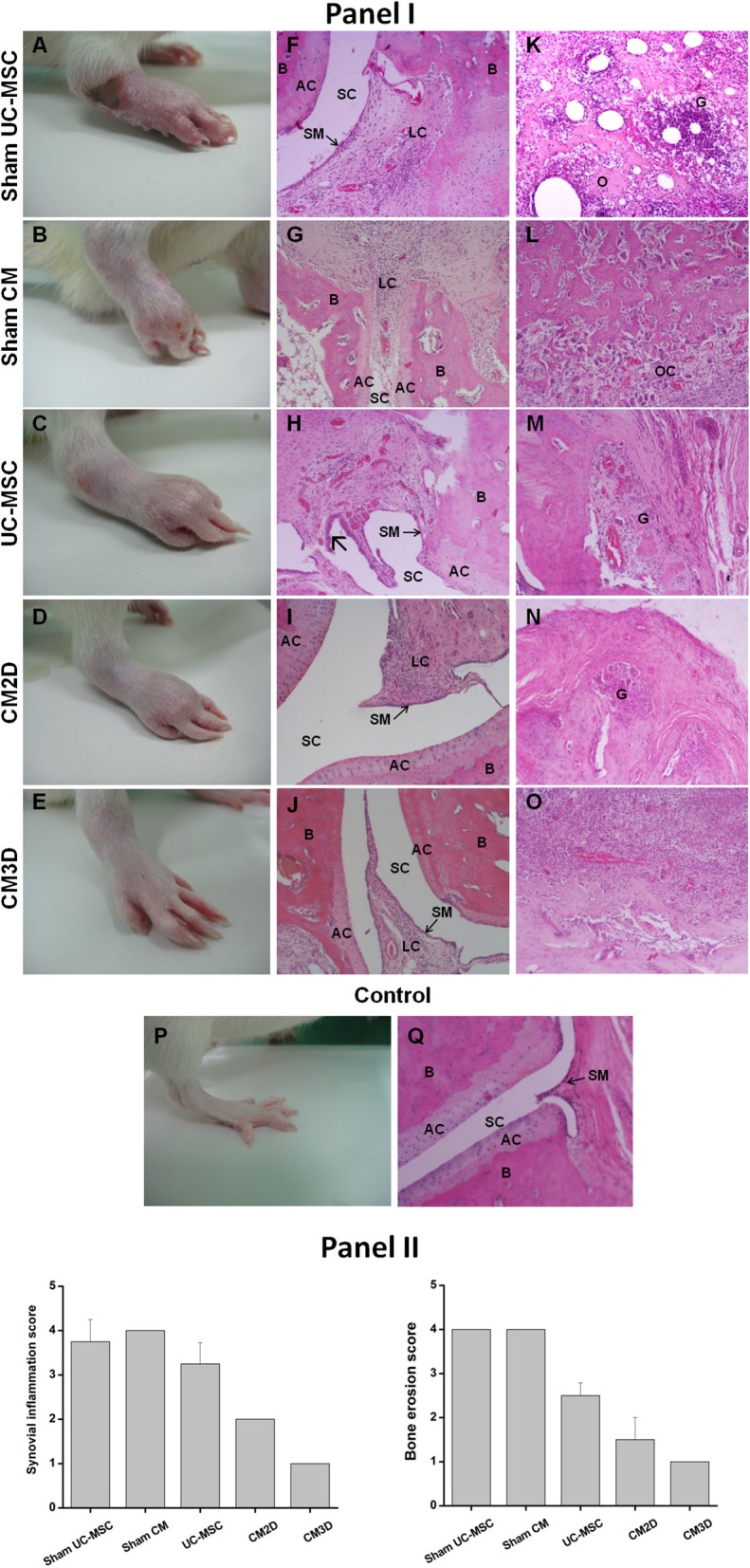
CM3D attenuates tissue destruction in adjuvant-induced arthritis (AIA) rats. (**Panel I**) Representative images of induced hind paws obtained from rats treated with UC-MSCs, CM3D, and CM2D, and the respective controls (Sham), on day 57 after arthritis induction. Representative images of non-induced hind paw (control) are also shown. **(A–E)** Macroscopic images of induced hind paws. **(F–O)** Representative photomicrographs from rat paw histological sections stained with H&E revealing signs of **(F–J)** synovitis and/or **(K–O)** osteolysis. Specifically: **(F)** sham UC-MSC histological sections show blood vessel congestion and severe synovitis with infiltration of lymphoid cells. **(K)** A pronounced osteolysis is also observed with the presence of granuloma, extensive neutrophil accumulation and bone necrosis. **(G)** Sham CM condition reveals synovial membrane with intense infiltration of inflammatory cells and capillary vessels congestion and **(L)** extensive number of osteoclasts. **(H,M)** UC-MSC-treated animals present milder granulomatous lesions than respective sham and hyperplasia of the synovial membrane (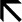). **(I,J,N,O)** CM-treated animals exhibit lower inflammation and bone destruction level. **(I)** CM2D image shows infiltration of lymphoid cells in a lower extent than respective sham and **(N)** some degree of osteolysis. **(J,O)** CM3D image shows a synovial membrane with less blood vessels and cell infiltration than respective sham, being the changes located in the extra-articular space. **(P,Q)** Control paw from a naïve animal shows normal histological features, with no evidence of inflammation and with intact synovial membranes. (**Panel II**) Histological scores for synovial inflammation and bone erosion. Data represent the mean ± SEM. *n* = 3 for control; *n* = 4 for sham groups; *n* = 6 for all other groups. AC, articular cartilage; B, bone; G, granuloma; LC, lymphoid cells; O, osteolysis; OC, osteoclasts; SC, synovial cavity; SM, synovial membrane.

The results of a more in-depth analysis of AIA manifestations at day 57 are depicted in Figure 6. Representative hind-paw photos of each experimental group clearly show that untreated animals, belonging to control (Sham) groups, still presented moderate-to-severe swelling encompassing the ankle, foot, and digits, with multiple foci of necrosis, inflammation, secondary infection, and joint deformity (Figures 6A,B). Notably, CM3D-treated animals exhibited only minimal paw swelling, with no signs of lesion (Figure 6E) when compared to CM2D- (Figure 6D) and UC-MSC-treated (Figure 6C) animals that still presented a moderate degree of swelling and moderate-to-negligible signs of lesion. Accordingly, the histopathological analysis of control (Sham) animals showed extensive osteoclastic activity along with the presence of granulomas, affecting the limits of the cartilage and bone tissues (Figures 6F,G,K,L). Bone necrosis appeared mainly in the periphery where numerous osteoclasts were noticed (Figures 6K,L). Synovitis was detected in all cases which represented the initial phase of RA (Figures 6F,G). No significant differences were observed between the two Sham groups (i.a. vs. i.p.). In turn, UC-MSC-treated animals still showed some signs of inflammation as seen by the presence of granulomas in the osteolytic area (Figure 6M) and of hyperplasic synovium membrane (Figure 6H). Finally, both animals treated with CM2D and CM3D presented small foci of synovitis with almost well-defined bone and cartilage tissue stratification (Figures 6I,J). Moreover, the osteolysis degree observed in CM3D-treated animals (Figure 6O) is lower than that of animals treated with CM2D (Figure 6N), in which granulomatous lesions are still present.

## Discussion

MSCs are known to modulate tissue regeneration through trophic effects exerted by secreted cytokines and growth factors. In fact, and contradicting the dogma that cells need to be physically present to induce regeneration through mechanisms involving homing, engrafting, and secretion of trophic factors in response to local stimuli, our previous results have unlocked the possibility of using the MSC secretome as active substance for therapeutic formulations. By recreating a more physiological environment within our 3D culture system, characterised by tissue-like cell-to-cell and cell-to-ECM interactions, as well as the presence of stress signals discharged by MSCs within the core of self-aggregated spheroids (e.g., hypoxic and famine), we were able to mimic many stimuli found within the lesion niche.

In this work, we aimed at demonstrating the viability of applying this 3D-priming strategy to improve the efficacy of the resulting UC-MSC secretome for counteracting the signs caused by inflammatory arthritis. A comparative analysis of CM3D and CM2D proteomes, comprehending a significant set of relevant trophic factors, corroborated our previous finding that our 3D conditions promoted different trophic profiles. Within our pool of trophic factors, CM3D was marked by synthesis of mainly IL-10, LIF, FGF-2, I-309, GM-CSF, eotaxin, and MIP-1α, and to a lower extent G-CSF, PDGF-BB, 6CKine, and SCF. In turn, CM2D was characterised by significantly higher expression of mainly IL-6, MCP-1, and IL-21.

The impact of such differences was validated *in vitro* where CM3D showed significantly higher motogenic activity over chondrocytes when compared to CM2D. This could be explained by e.g., relatively higher CM3D expression of e.g., IL-10, FGF-2, and PDGF-BB (33–35). IL-10 was found to directly protect chondrocytes *in vitro* through the inhibition of NOS2 and MMP-3 expression. In turn, FGF-2 and PDGF-BB are potent mitogens for articular chondrocytes which have also been found to promote chondrogenic differentiation (33, 35, 39).

Different paracrine activities between CM3D and CM2D were further validated *in vitro* by evaluating their relative capacities to induce GAG synthesis. This time CM2D proved to be a more potent inducer of GAG synthesis than CM3D, which could be justified by the relatively higher CM2D expression of IL-6. IL-6 was found to induce IL-1β-related collagen and GAG biosynthesis and to stimulate cartilage matrix production *in vitro* (37, 40).

Although *in vitro* indications suggested differential capacities of CM3D and CM2D to induce important events connected to tissue regeneration, their impact in the specific context of AIA could not be directly extrapolated. For example, although apparent benefits should be drawn from FGF-2-induced chondrocyte motility in a OA context, FGF-2 expression in RA patients has been closely associated with disease severity (41). Recently, FGF-2 was even shown to cooperate with IL-17 in the pathogenesis of autoimmune arthritis (42). Similarly, while GAG production promoted by IL-6 is necessary for the regeneration of functional cartilage in osteoarthritic patients, self-antigenic GAGs were found to provoke autoimmune dysfunctions that involve the expansion of GAG-binding infiltrates, thus aggravating inflammatory conditions in RA (43). The amelioration effects of either CM3D or CM2D on arthritic signs would therefore be dependent on how the overall synergistic activity within each of their trophic factor compositions would interrelate with the AIA environment.

Ultimately the results showed that CM3D has a clearly superior capacity for both, avoiding and ameliorating AIA manifestations *in vivo* when compared to CM2D or even UC-MSCs. CM3D treatment was able to both prevent and revert all major signs of AIA, including complete avoidance of necrotic foci around the joints, acute and chronic inflammation, joint deformity and secondary infection.

Mechanisms behind CM3D activity can also be extrapolated based on secretome profile features resulting from our comparative analysis. Mainly IL-10, a recognised potent anti-inflammatory type II cytokine, plays a central role in limiting host immune response to pathogens. Dysregulation of IL-10 is associated with enhanced immunopathology in response to infection as well as increased risk for development of many autoimmune diseases (34, 44, 45). IL-10 was found to be produced by innate cells, as well as CD4^+^ CD25^−^ Foxp3^−^ and CD4^+^ CD25^+^ Foxp3^+^ Tregs using a *Leishmania* chronic lesion model, which is consistent with our previous observations that UC-MSCs enhanced CD4^+^ CD25^+^ Foxp3^+^ Tregs in response to a AIA environment *in vivo* (20, 46). IL-10 was also shown to limit the inflammasome (NLRP3)-driven arthritic disease course and associated structural damage in an AIA model (47). Furthermore, reversion of arthritis by IL-10 was not limited to AIA. IL-10 produced by B cells was also found to be crucial for the suppression of Th17/Th1 responses, induction of T regulatory type 1 cells and the reduction of collagen-induced arthritis (CIA)-related signs, many common to AIA (48). The recognised IL-10 distinctive capacities to downregulate the production of pro-inflammatory cytokines meant that it has been regarded as a potential therapeutic agent for the treatment of arthritis (49). Besides IL-10, the highly expressed leukaemia inhibitory factor (LIF) can augment the immunosuppression capacity of CM3D through further induction of Tregs (50–52). LIF may also play an important role in regulating the neural-immune system interaction during early acute inflammatory stages of the disease and the subsequent healing and restitution process (53). Concomitantly, other CM3D highly expressed factors such as FGF-2 and I-309, have been found to be involved in different aspects of tissue regeneration. FGF-2 through mitogenic and motogenic activities over chondrocytes (39) and I-309 and SCF through promotion of angiogenesis (27, 54). Yet other factors produced in CM3D, such as SCF and G-CSF, have been found to support haematopoiesis and recruiting of other CD 34^−^ endogenous MSCs to aid in regeneration (18, 28, 55). The remarkably low incidence of secondary infection signs in animals treated with CM3D could have been due to the relatively higher expression of cytokines such GM-CSF, 6CKine, and eotaxin. Although these factors may contribute to pathogenic inflammatory infiltrate, GM-CSF for e.g., has even been used recently as primary immuno-target for treatment of specific groups of RA patients (56), their synergistic roles in stem cell stimulation and eosinophil recruitment may confer advantages in a AIA context; especially when coupled with the expression of anti-inflammatory cytokines like IL-10 and LIF and within an environment characterised by opportunistic secondary infection. Thus, in our experimental conditions, GM-CSF, together with other cytokines usually associated with RA pathogenesis, but with capacities to attract lymphocytes with distinct phenotypes, like MIP-1α, MIP-1β, 6CKine, and RANTES, could be modulating specific T-cell functions in favour of a wider host defence. In addition, eotaxin could be attracting eosinophils to provide further defence against infectious agents while producing antihistamines (57–63).

Finally, differences seen between CM3D and CM2D could also be explained by a distinctive pro-inflammatory character of CM2D, as patented by a comparatively higher expression of inflammatory MCP-1, IL-6, and IL-21 without counterbalancing expression of anti-inflammatory cytokines. Both MCP-1 and IL-6 have for long been found to be highly expressed in the synovial fluid of RA patients (64). MCP-1 and IL-6 have been consistently found to play critical roles in the development of AIA signs in several animal models (31, 37, 65). Post-onset treatment of AIA using endogenous MCP-1 inhibitors improved clinical signs of arthritis and histological scores measuring joint destruction, synovial lining, macrophage infiltration, and bone erosion (66). In turn, IL-6 is synthesised in response to many stimuli, including IL-1β which is widely implicated in the pathogenesis of RA (36, 40). More recently, IL-21 has been found to be involved in several mechanisms related to RA pathogenesis being able to activate T cells, B cells, monocytes/macrophages and synovial fibroblasts through activation of JAK-STAT, MAPK, and PI3K/Akt signalling pathways, ultimately promoting osteoclastogenesis (32).

Overall the results demonstrate the viability of applying this 3D-priming strategy to improve the efficacy of the resulting UC-MSC secretome for counteracting the manifestations caused by inflammatory arthritis. On the path to simplify MSC-based therapeutic formulations more studies will now follow to discriminate what components within CM3D are exerting the observed protective and therapeutic activities. This will involve a concerted action applying multi-faceted analyses involving exosome scrutiny, proteomics, metabolomics as well as epigenomics and miRNA regulomics. A more difficult task will be to define precise synergistic relationships between the different actors within the formulations and predict their synergistic effect within different disease environments. Nevertheless, we believe a novel path has been unleashed, involving the use of well-defined paracrine actors, instead of physical cells, as active substances for “off-the-shelf” *Advanced Therapy Medicinal Products* (ATMP).

## Author Contributions

JM, SS, and JS developed the study concept and the study design. SC, MG, JR, and MC performed the experiments and data collection. SC, JR, and RB performed the data analysis and interpretation under the supervision of JM, SS, and JS. JM, SC, JR, SS, and JS drafted the manuscript. RB, PC, and HC provided critical revisions. All authors approved the final version of the manuscript for submission.

### Conflict of Interest Statement

The authors declare that the research was conducted in the absence of any commercial or financial relationships that could be construed as a potential conflict of interest.
